# Multi-centre evaluation of the speed-oligo Mycobacteria assay for differentiation of *Mycobacterium *spp. in clinical isolates

**DOI:** 10.1186/1471-2334-11-353

**Published:** 2011-12-19

**Authors:** Sabine Hofmann-Thiel, Laziz Turaev, Tarig Alnour, Lore Drath, Maria Müllerova, Harald Hoffmann

**Affiliations:** 1 IML red GmbH & synlab Gauting, Supranational Reference Laboratory of Tuberculosis, c/o Asklepios Fachkliniken, Robert-Koch-Allee 2, Gauting, Germany; 2 National Reference Laboratory of Tuberculosis, Tashkent, Uzbekistan; 3 Department of Microbiology, Alzaiem Alazhari University, Khartoum North, Sudan; 4 Bioscientia Institute of Medical Diagnostics GmbH, Ingelheim, Germany; 5 KLINLAB, Departments of Microbiology and Molecular Biology TB, synlab Prague, Prague, Czech Republic

## Abstract

**Background:**

A new DNA line probe assay (Speed-oligo Mycobacteria, Vircell) has been launched for rapid differentiation of *Mycobacterium *spp. from cultures. Compared to other line-probe assays, Speed-oligo Mycobacteria covers a relatively limited spectrum of species but uses a simpler and faster dip-stick technique. The present multi-centre, multi-country study aimed at evaluating the utility and usability of Speed-oligo Mycobacteria in routine mycobacteriology diagnostics. Results from Speed-oligo Myobacteria were compared to those from Genotype CM (HAIN lifescience, Nehren, Germany), another line-probe assay.

**Methods:**

Speed-oligo Mycobacteria assay was performed in three main steps: 1) DNA extraction from cultured material 2) PCR amplification of the target gene and an internal control and 3) hybridization of the PCR products to specific probes by means of a dip-stick.

**Results:**

Two hundred forty-two clinical isolates were recovered from consecutive positive mycobacterial cultures at two German (IML Gauting, Bioscientia Ingelheim), one Czech (KLINLAB Prague), and at a Sudanese (Khartoum) laboratory. All *Mycobacterium *species covered by the assay were reliably recognized. The rate of false positive results was 1.2% and concerned only the species *M. marinum *and *M. peregrinum*. The identification rate, i.e. the proportion of isolates which was correctly differentiated to the level of species or complex by the assay, differed significantly among laboratories being 94.9%, 90.7%, and 75.0% at the study sites IML Gauting, KLINLAB Prague and Bioscientia Ingelheim, respectively. This difference was caused by different spectra of NTM species encountered by the laboratory centres in daily routine diagnostics.

**Conclusions:**

Speed-oligo Mycobacteria assay was proved a rapid and easy-to-perform alternative to conventional line-probe assays. The assay showed excellent sensitivity with regard to identification of genus *Mycobacterium *and species/complexes covered by the test. However, due to its relatively limited spectrum of taxa, a varying proportion of NTM may not be identified by the assay in daily diagnostics demanding further analyses. The only significant shortcoming in terms of specificity was the misidentification of the clinically relevant species *M. marinum*.

## Background

Whereas tuberculosis (TB) still represents one of the major public health problems in the non-industrialized world, particularly in Africa, South/East-Asia and the countries of the former Soviet-Union [1], its prevalence is steadily declining in the fully industrialized world. Here however, infections caused by non-tuberculous mycobacteria (NTM) are becoming more and more relevant in both, incidence and morbidity, partly due to the spread of iatrogenic immunosuppression [2,3]. Since management, treatment, and infection control measures differ dramatically between TB and NTM infections, a rapid and accurate differentiation between these entities is mandatory as soon as a mycobacterial culture turns positive. Additionally, rapid identification of the most frequent NTM species is desirable helping the clinical doctor to assess the relevance of the laboratory findings.

Most molecular genetic tests differentiating mycobacterial species fulfil this postulation including sequencing of target genes like 16S rRNA gene (rDNA) [4,5], *hsp65 *gene [6] or 16S-23S intergenic transcribed sequences (ITS) [7] and commercial tests based on gene probes. Among the commercial tests, the gene probe assay AccuProbe (Gen-Probe, San Diego) was the first one to be developed. The limitation of this fast single-probe test is the relatively small spectrum of *Mycobacterium *spp. [8,9]. With the introduction of DNA line probe assays (LPA), the simultaneous differentiation of several mycobacterial species in one single test has become possible. INNO-LiPA Mycobacteria v2 (Innogenetics, Ghent, Belgium) and the GenoType Mycobacterium CM and AS (HAIN Lifesciences, Nehren, Germany) are currently among the most widely used LPAs. They have been evaluated extensively with different panels of mycobacterial species [8,10,12-15] and proved easy-to-interpret, sensitive, specific, cost-effective, and in excellent concordances with independent reference methods such as sequence analyses of housekeeping genes. On the other hand, they require higher turnaround- as well as hands-on-times than for example the Accuprobe system.

Recently, a new LPA for the differentiation of mycobacterial species was introduced. The Speed - oligo Mycobacteria^® ^assay (Vircell, Santa Fé Granada, Spain) uses PCR of 16S-23S ITS target sequences and subsequent hybridization to immobilized probes by means of a fast and easy dip stick technique. It detects up to 12 different taxa represented by six specific probes on the strip covering a smaller spectrum than other LPAs. A recent study assessed the performance of the Speed-oligo Myobacteria assay from cultures in a single laboratory in Spain [16] proving good concordance (97.2%) to reference methods.

The spectrum and frequency of NTM may vary significantly between regions in Europe and the world [17-20] having potential implications on the validity of the assay in daily diagnostics. Therefore, a multi-centre and multi-country approach was initiated evaluating Speed-oligo Mycobacteria for differentiation and identification of *Mycobacterium *spp. from cultures in different settings of routine mycobacteriology diagnostics.

## Methods

### Clinical isolates

Clinical isolates (n = 242) included in this study were recovered from consecutive positive mycobacterial cultures originating from routine diagnostic services at four laboratory centres after N-acetyl-cystein (NALC)-NaOH decontamination. At the European centres IML Gauting, Bioscientia Ingelheim (both Germany) and KLINLAB Prague (Czech Republic), 59 (February-June 2010), 60 (June-September 2009), and 75 (February-July 2010) isolates, respectively, were obtained within the indicated time periods. At the laboratory in Khartoum (Sudan), 53 consecutive sputum samples from smear positive patients (September-December 2009) were decontaminated using (NALC)-NaOH [21], re-suspended in 1.0 ml phosphate buffer (pH 6.8) and shipped to the IML Gauting for growth detection using solid and fluid media (MGIT™, Becton-Dickinson, Heidelberg, Germany). Of these, 48 MGIT cultures turned positive with acid-fast bacilli and were tested at the IML Gauting by the Sudanese co-author.

### Reference methods

All isolates were differentiated to the level of species by routine differentiation methods. At all centres, Genotype CM was the firstly used differentiation assay. In case of a *Mycobacterium tuberculosis *complex (MTBC) specific band pattern, Genotype MTBC was performed. If Genotype CM yielded another species-specific band pattern the resulting NTM species was assumed as correct--provided that the identification result was in agreement with growth characteristics and morphology of the isolate-and no further tests were performed. If only genus *Mycobacterium *was identified, Genotype CM was followed by Genotype AS at study sites Bioscientia Ingelheim and KLINLAB Prague, and if necessary partial 16S rDNA sequencing, or directly subjected to sequencing at study site IML Gauting. Two *Nocardia *spp. were not differentiated to the level of species. In cases of discrepant results by Genotype and Speed-oligo assays, the isolate was re-assessed by sequencing for definite species identification.

### Speed-oligo mycobacteria assay

For DNA extraction, a loop-full of bacteria from Loewenstein-Jensen (LJ) slant culture or bacterial sediments from 1 ml of liquid media were re-suspended in 300 μl Vircell sample solution and incubated at 95°C for 60 min. After centrifugation for 5 min at 12,000 × *g*, the lysates were directly used for PCR or stored frozen at − 20°C. For PCR amplification, 15 μl of reconstituted ready-to-use PCR mix (provided with the kit) was added to PCR tubes in a DNA-free and PCR amplicon-free pre-amplification area. Then, 10 μl of DNA sample was added and PCR amplification (1 min 92°C, 40 cycles consisting of 10 s 92°C, 30 s 55°C, 30 s 72°C, 1 min 72°C) was started. The PCR product was denaturated for 1 min at 95°C and immediately cooled on ice for no more than 1 min. Then, 5 μl of PCR product was added to 40 μl of pre-heated (55°C) running solution in a 1.5 ml tube (post-amplification area). The Speed-oligo strip was immediately inserted and results could be interpreted after 5 min of incubation at 55°C. Results were considered valid, when both internal control lines (PCL, product control line; PCRCL, PCR control line) were positive. Speed-oligo Mycobacteria includes six specific probes (TL1 to TL6) allowing for the identification of members of the *M. tuberculosis *complex (MTBC) (TL4) and frequently encountered NTMs, i.e. *M. avium*/*M. intracellulare*/*M. scrofulaceum *group (TL5), *M. chelonae*/*M. abscessus *complex (TL1), *M. kansasii *(TL3)*, M. gordonae *(TL2), and *M. fortuitum *(TL6), each of them yielding individual patterns (Table 1). According to the manufacturer, members of the MTBC can show weak TL3 signals in addition to the MTBC specific TL4 probe. A genus specific probe (TL7) additionally allowed for the identification of the genus *Mycobacterium*.

**Table 1:** Interpretation of Speed-oligo Mycobacteria results

Microorganism	**Specific line(s*)***^a^
*M. chelonae */*M. abscessus *complex	TL1

*M. gordonae*	TL2

*M. kansasii*	TL3

*M. tuberculosis *complex (*M. tuberculosis*, *M. africanum, M. bovis*, *M. microti*)	TL4 or TL4 + TL3

*M. avium */*M. intracellulare */*M. scrofulaceum group*	TL5

*M. fortuitum*	TL6

*Mycobacterium *genus	TL7

^a^Depending on the mycobacterium present in the sample, signals will be observed in indicated lines. According to the manufacturer, TL4 and TL4 + TL3 are interpreted as *M. tuberculosis *complex.

## Results

Two hundred forty-two clinical isolates were included in the study (Table 2). They originated from four laboratory centres, two in Germany, one in the Czech Republic, and one in the Sudan. The reference methods used identified 77 isolates (31.8%) as members of the MTBC, 162 (66.9%) as NTM and three (1.2%) as species of related genera (two *Nocardia *spp., one *Tsukamurella pulmonis*).

**Table 2:** Multi-centre evaluation of Speed-oligo Mycobacteria and comparison to Genotype CM

*Mycobacterium species ^a^*	*no. of Strains*		*Speed-oligo results*	*Genotype CM results ^d^*
			
		*Bands^b^*	*Identification^c^*	
**IML Gauting, Germany**	**59**			

*M. tuberculosis*^e^	13	(3), 4, 7	*M. tuberculosis *complex	*M. tuberculosis *complex

*M. avium*	14	5, 7	MAIS group	*M. avium*

*M. intracellulare*	9	5, 7	MAIS group	*M. intracellulare*

*M. intracellulare*	1	5, 7	MAIS group	*Mycobacterium *spp.

*M. kansasii*	7	3, 7	*M. kansasii*	*M. kansasii*

*M. kansasii*	1	3, 7	*M. kansasii*	*Mycobacterium *spp.

*M. abscessus*	1	1, 7	*M. chelonae/M. abscessus *complex	*M. abscessus*

*M. chelonae*	1	1, 7	*M. chelonae/M. abscessus *complex	*M. chelonae*

*M. gordonae*	7	2, 7	*M. gordonae*	*M. gordonae*

*M. fortuitum*	2	6, 7	*M. fortuitum*	*M. fortuitum*

*M. peregrinum*	1	(6), 7	*M. fortuitum*	*M. peregrinum*

*M. xenopi*	1	7	*Mycobacterium *spp.	*M. xenopi*

*Tsukamurella pulmonis*	1	-	no *Mycobacterium *spp.	Gram+ bacteria, high G+C content

**Bioscientia Ingelheim, Germany**	**60**			

*M. tuberculosis*^e^	13	(3), 4, 7	*M. tuberculosis *complex	*M. tuberculosis *complex

*M. bovis *BCG^e^	1	(3), 4, 7	*M. tuberculosis *complex	*M. tuberculosis *complex

*M. avium*	4	5, 7	MAIS group	*M. avium*

*M. intracellulare*	6	5, 7	MAIS group	*M. intracellulare*

*M. scrofulaceum*	1	5, 7	MAIS group	*M. scrofulaceum*

*M. kansasii*	1	3, 7	*M. kansasii*	*M. kansasii*

*M. chelonae*	3	1, 7	*M. chelonae/M. abscessus *complex	*M. chelonae*

*M. gordonae*	14	2, 7	*M. gordonae*	*M. gordonae*

*M. fortuitum*	2	6, 7	*M. fortuitum*	*M. fortuitum*

*M. celatum*	4	7	*Mycobacterium *spp.	*Mycobacterium *spp.

*M. flavescens*	1	7	*Mycobacterium *spp.	*Mycobacterium *spp.

*M. malmoense*	1	7	*Mycobacterium *spp	*M. malmoense*

*M. montefiorense*	1	7	*Mycobacterium *spp.	*Mycobacterium *spp.

*M. peregrinum*	1	6, 7	*M. fortuitum*	*M. peregrinum*

*M. simiae*	3	7	*Mycobacterium *spp.	*Mycobacterium *spp.

*M. xenopi*	2	7	*Mycobacterium *spp.	*M. xenopi*

*Nocardia spp*.	2	-	no *Mycobacterium *spp.	n.d.

**KLINLAB Prague, Czech Rep**.	**75**			

*M. tuberculosis*^e^	26	(3), 4, 7	*M. tuberculosis *complex	*M. tuberculosis *complex

*M. bovis *BCG^e^	1	(3), 4, 7	*M. tuberculosis *complex	*M. tuberculosis *complex

*M. avium*	13	5, 7	MAIS group	*M. avium*

*M. intracellulare*	5	5, 7	MAIS group	*M. intracellulare*

*M. kansasii*	3	3, 7	*M. kansasii*	*M. kansasii*

*M. abscessus*	1	1, 7	*M. chelonae/M. abscessus *complex	*M. abscessus*

*M. chelonae*	1	1, 7	*M. chelonae/M. abscessus *complex	*M. chelonae*

*M. gordonae*	9	2, 7	*M. gordonae*	*M. gordonae*

*M. fortuitum*	9	6, 7	*M. fortuitum*	*M. fortuitum*

*M. lentiflavum*	2	7	*Mycobacterium *spp.	*Mycobacterium *spp.

*M. marinum*	1	3, 7	*M. kansasii*	*M. marinum*

*M. xenopi*	4	7	*Mycobacterium *spp.	*M. xenopi*

**Khartoum, Sudan**	**48**			

*M. tuberculosis*^e^	16	(3), 4, 7	*M. tuberculosis *complex	*M. tuberculosis *complex

*M. tuberculosis*^e ^*+ M. fortuitum*	7	(3), 4, 6, 7	*M. tuberculosis *complex + M. fortuitum	*M. tuberculosis *complex + *M. fortuitum*

*M. fortuitum*	25	6, 7	*M. fortuitum*	*M. fortuitum*

***TOTAL***	***242***			

^a^Differentiation to species level by use of Genotype CM, Genotype AS, Genotype MTBC or sequencing (16S rDNA or *hsp65 *gene).

^b^Numbers in brackets indicate weaker staining.

^c^MAIS group: *M. avium M. intracellulare M. scrofulaceum *group.

^d^Identification results by Genotype CM are shown. n.d.: not determined.

^e^All MTBC isolates yielded weak TL3 signals in addition to TL4

### Sensitivity and false results

Speed-oligo Mycobacteria yielded valid results for all 242 study isolates. Two hundred seventeen isolates (89.7%) belonged to species covered by specific Speed-oligo probes. They yielded the expected patterns and showed complete concordance to the reference method (Table 2). Twenty-five isolates belonged to species not covered by specific Speed-oligo probes. Twenty-two of them (88.0%) yielded correct results, i.e. 19 NTM isolates were identified as *Mycobacterium *spp. and three isolates from other genera did not react with any TL probe.

Three NTM isolates (1.2%, one from each European study site) yielded unspecific Speed-oligo signals leading to misidentifications: two *M. peregrinum *isolates showed a weak reaction with TL6 probe and were thus misidentified as *M. fortuitum*; one *M. marinum *isolate reacted with TL3 probe and was thus misidentified as *M. kansasii*.

Overall, the sensitivity of Speed-oligo Mycobacteria--defined as the proportion of correct identifications of isolates belonging to taxa for which the assay possesses specific probes--was 100% for the genus specific probe TL7 (239 of 239 isolates) and 100% for the species/complex specific probes TL1-6 (217 of 217 isolates). The rate of false results--defined as the proportion of test outcomes leading to misidentifications of species--was 1.2% for species/complex specific probes (3 of 242 isolates).

### Identification rate

Speed-oligo Mycobacteria identified the species/complex of 217 out of 242 clinical isolates (89.7%). The spectrum of mycobacterial species and consequently the outcome of Speed-oligo Mycobacteria varied significantly from study site to study site (Table 2). At IML Gauting and KLINLAB Prague, 95.5% and 85.4%, respectively, of NTM isolates belonged to species which were covered by Speed-oligo probes. At Bioscientia Ingelheim this proportion was only 70%. Among Sudanese samples, it was 100% since the only NTM species found was *M. fortuitum *which is covered by Speed-oligo Mycobacteria. Consequently, the Speed-oligo identification rate, i.e. the proportion of clinical isolates differentiated to the level of species or complex, was relatively poor at Bioscientia Ingelheim (45 of 60 clinical isolates, 75.0%), good at KLINLAB Prague (68 of 75, 90.7%) and at IML Gauting (56 of 59, 94.9%) but excellent for the Sudanese isolates (48 of 48, 100.0%).

### Comparison to results of Genotype CM

At all study sites, Genotype CM was used as primary method for differentiation as soon as a mycobacteriological culture turned positive. Therefore, Genotype CM results were available for all study isolates and allowed us to compare them directly to Speed-oligo results (Table 2). Of 77 MTBC isolates, all yielded identical results with both assays. Further differentiation to the species level (76 MTB, one *M. bovis *BCG) confirmed the identifications by both assays.

Concordance of the assays was 98.7% with respect to 140 NTM isolates belonging to species covered by Speed-oligo Mycobacteria except the following two discrepancies: Genotype CM did not identify the species of one *M. intracellulare *and one *M. kansasii *isolate and differentiated them only to the level of the genus. Noteworthy, all but these two isolates were differentiated to the species level by Genotype CM whereas 58 of 140 isolates were only assigned to groups or complexes of species by Speed-oligo Mycobacteria.

Twenty-two NTM isolates belonged to species not included in Speed-oligo Mycobacteria. Eleven of them were differentiated to the species level by Genotype CM yielding *M. xenopi *(n = 7), *M. malmoense *(n = 1), *M. marinum *(n = 1), and *M. peregrinum *(n = 2). The remaining NTM isolates belonged to the taxa *M. celatum*, *M. flavescens*, *M. montefiorense*, *M. lentiflavum *and *M. simiae *and were identified neither by Genotype CM nor by Speed-oligo Mycobacteria.

Overall, Genotype CM would have been theoretically able to differentiate 228 out of 242 isolates. However, it differentiated 226 of them correctly resulting in a sensitivity of 98.7% (Speed-oligo: 100%). None of the Genotype CM tests performed lead to misidentification of the species being better than that of Speed-oligo Mycobacteria. Genotype CM yielded identification rates of 97.3% (73 of 75 isolates), 94.9% (56 of 59 isolates), and 81.7% (49 of 60 isolates), at KLINLAB Prague, IML Gauting, and Bioscientia Ingelheim, respectively.

### Evaluation of work processes and time required

Speed-oligo Mycobacteria has been assessed as easy to perform by the laboratory staff at each of the European centres. In particular, the availability of a ready-to-use PCR mix as well as the dip-stick technique was considered a significant simplification of laboratory work processes. At the IML Gauting the total assay time of Speed-oligo Mycobacteria (when handling five strains) was measured to 3 h composed of 1 h 10 min for DNA extraction, 10 min for pre-PCR steps, 1 h 30 min for PCR amplification and 10 min for hybridization by dip-stick technique. Cumulative hands-on-time was estimated to 25 min.

## Discussion

Speed-oligo Myobacteria has been developed for the rapid differentiation of mycobacterial species from cultures. Its major advantage consists in its simple dip stick technique which is directly applied to denaturized amplification products. The dip stick procedure is easy to perform requiring only two pipetting steps thereby decreasing the hands-on time as well as the risk of cross-contaminations. Results can be obtained within about 3 h with a total hands-on time of 25 min. By comparison, the line probe assays Genotype Mycobacteria CM/AS and INNO-LiPA Mycobacteria require a total time of 5-6 h with hands-on times of about 2 h [8,13,22].

One of the major limitations of Speed-oligo Mycobacteria is that it differentiates only a relatively small spectrum of *Mycobacterium *species. This gives rise to lower identification rates compared to similar LPAs like Genotype CM depending on regional differences regarding the spectrum of encountered NTM species. Among our four study centres, identification rates varied significantly. We observed the best identification rate with the Sudanese samples due to the fact that *M. fortuitum *was the only NTM species found. Generally, *M. fortuitum *is considered one of the most frequent contaminants recovered from clinical [23] and environmental specimens [24] in Africa. Our Sudanese sputa were transported for approximately 1 week before they reached our laboratory. Probably, some of them were overgrown by *M. fortuitum *during shipment biasing the species distribution in this panel. Considering European laboratories, Speed-oligo Mycobacteria showed a good identification rate of 94.9% with isolates from IML Gauting being in the range reported for INNO LiPA or Genotype CM assays in the literature (88-96%) [13,22,25] and identical to that shown for Genotype CM in the present study. By contrast, identification rates of Speed-oligo Mycobacteria decreased to 90.7% at KLINLAB Prague and 75% at Bioscientia Ingelheim while the rates of Genotype CM were significantly better at these sites (97.3% and 81.7%). This was mainly due to the species *M. xenopi *which is not covered by Speed-oligo Mycobacteria. *M. xenopi *is increasingly encountered throughout the world [17,18,20] reaching relative frequencies of up to 26% of NTM isolates [20]. In order to meet the demands of an identification system covering the most frequent mycobacterial species, incorporation of a *M. xenopi *specific probe should be considered by the manufacturer.

Compared to other line-probe assays, Speed-oligo Mycobacteria differentiates most of the species only to a level of complexes or groups. Current guidelines do not demand the differentiation of the species *M. avium *and *M. intracellulare*, although the discrimination to *M*. *scrofulaceum *would be desirable [26]. However, the differentiation between *M. chelonae *and *M. abscessus *is considered essential for decisions on the optimal regimen if treatment is necessary.

Overall, all species included in the Speed-oligo Mycobacteria assay were reliably identified and yielded the expected patterns. Among these isolates, Genotype CM failed to identify one *M*. *intracellulare *and one *M. kansasii *isolate. The latter showed an unspecific Genotype pattern (bands 1, 2, 3, 10, 12, 13) and was attributed to *M. kansasii *sequevar VI-3 by sequence analysis of the 16S rDNA; this sequevar of *M. kansasii *had been previously described to be missed by Genotype CM [14]. Partial 16S rDNA sequencing of the *M. intracellulare *isolate showed identity to *M. avium *complex ATCC 35770 strain sequences corresponding to *M. intracellulare *serovar 18/MAC D [27]. Phylogenetically, *M. intracellulare *serovar 18 forms a rather unique 16S rDNA pattern [28] and appears to be only rarely isolated from human specimens [29]. To our best knowledge, there are no published data regarding performance of Genotype CM.

Speed-oligo Mycobacteria yielded only three out of 242 isolates (1.2%) with false positive reactions. Cross-reaction of *M. peregrinum *with TL6 has not been described so far but can be explained by the relatedness of *M. peregrinum *and *M. fortuitum *which both belong to the *M*. *fortuitum *complex. Cross-reaction of *M. marinum *with the TL3 probe is in concordance with the observation by Quezel-Guerraz et al. [16] and is already mentioned in the user's manual by Vircell Microbiologists. Potential misidentification of *M. marinum *as *M. kansasii *is certainly the major shortcoming of the assay. *M. marinum *regionally reaches relative frequencies of up to 2% of NTM isolates [17,19]. Although infection due to *M. marinum *clinically presents with characteristic cuteneous lesions, misidentification may cause confusion and unnecessary delay of definite diagnosis.

## Conclusions

Our aim was a multi-centre, multi-country evaluation of Speed-oligo Mycobacteria to differentiate *Mycobacterium *spp. from cultures. Two hundred forty-two clinical isolates obtained at four laboratory centres were included. The Speed-oligo Mycobacteria assay was found to represent a rapid and easy-to-perform alternative to conventional line-probe assays for laboratories that do not need to differentiate all NTM isolates to the species level. The test is very reliable recognizing species that are covered by specific probes on the dip-stick. The rate of false positive reactions among 242 isolates was as low as 1.2%. However, due to the more limited spectrum of species covered by the assay, a varying proportion of NTM in the diagnostic services may not be identified. In some settings the differentiation down to the species level might be required demanding additional analyses.

## Competing interests

The authors declare that they have no competing interests.

## Authors' contributions

S.H.-T. participated in the design of the study, supervised the laboratory work at the IML Gauting, analysed the data and drafted the manuscript; L.T. and T.A. did most of the experimental work at the IML Gauting; L.D. was responsible for testing of clinical isolates and data analysis at Bioscientia Ingelheim; M.M. was responsible for testing of clinical isolates and data analysis at KLINLAB Prague; H.H. supervised the study, participated in its design and the preparation of the manuscript. All authors read and approved the final manuscript.

## Pre-publication history

The pre-publication history for this paper can be accessed here:

http://www.biomedcentral.com/1471-2334/11/353/prepub
